# A network community structure similarity index for weighted networks

**DOI:** 10.1371/journal.pone.0292018

**Published:** 2023-11-29

**Authors:** Milad Malekzadeh, Jed A. Long

**Affiliations:** Department of Geography and Environment, Western University, London, ON, Canada; Marshall University, UNITED STATES

## Abstract

Identification of communities in complex systems is an essential part of network analysis. Accordingly, measuring similarities between communities is a fundamental part of analysing community structure in different, yet related, networks. Commonly used methods for quantifying network community similarity fail to consider the effects of edge weights. Existing methods remain limited when the two networks being compared have different numbers of nodes. In this study, we address these issues by proposing a novel network community structure similarity index (NCSSI) based on the edit distance concept. NCSSI is proposed as a similarity index for comparing network communities. The NCSSI incorporates both community labels and edge weights. The NCSSI can also be employed to assess the similarity between two communities with varying numbers of nodes. We test the proposed method using simulated data and case-study analysis of New York Yellow Taxi flows and compare the results with that of other commonly used methods (i.e., mutual information and the Jaccard index). Our results highlight how NCSSI effectively captures the impact of both label and edge weight changes and their impacts on community structure, which are not captured in existing approaches. In conclusion, NCSSI offers a new approach that incorporates both label and weight variations for community similarity measurement in complex networks.

## 1. Introduction

Due to their complex structure, the identification of communities continues to be an essential part of the analysis of networks [1]. Accordingly, measuring similarities between communities is a fundamental part of analysing community structure in different (usually related) networks [2–11]. Methods for assessing network community similarity can be employed to assess network models (e.g., comparing to known communities) [12, 13], comparing different states of the system (e.g., before and after an intervention), or time series analysis in networks [14].

Although several methods exist for quantifying network community similarity, such as the widely-used Jaccard index [2] and mutual information [15], they predominantly focus on community labels and neglect the intricate relationships encoded within edge weights. A critical drawback arises when a uniform metric treats dissimilarities in edges and their corresponding weights equivalently. For example, in a subway network, the cost of incorrectly clustering the central station (with a high number of edges and traffic flows due to its functionality) is the same as the cost of incorrectly clustering an intermediate station (with a low number of edges and traffic flows). This gap in the existing literature underscores the need for a novel approach that effectively incorporates edge weights into the community similarity calculation. Additionally, the application of current methods is not possible when comparing networks with different number of nodes.

We propose a novel network community structure similarity index (NCSSI) as a similarity index for comparing network communities. The formulation of the NCSSI is based on the concept of edit distance, whereby the cost of each node changing its community is computed based on their edges and corresponding weights. By taking this approach, the NCSSI provides an advantage over existing methods in that it is able to incorporate community labels, edges and their weights into a single measurement of community similarity. Employing simulated data and real-life New York Yellow Taxi flows data, we highlight how NCSSI compares to existing approaches and provides an alternative view of how similarity structure can be measured between communities.

The structure of the article is as follows: in section 2 we provide a general background on network structure, community detection, and similarity of communities. Then we introduce NCSSI and its implementation. In section 4 we experiment with NCSSI and simulated data in two different scenarios: on benchmarks to compare with existing similarity methods, and on benchmarks where we change the weights of random nodes to highlight the limitation of existing similarity methods. Then in section 5 we implement NCSSI on a real-life mobility dataset, New York Yellow Taxi flows data. In the discussion and conclusion, we discuss the advantages of the proposed method and the results, and we conclude the study.

## 2. Background/literature review

Many complex systems can be modeled by networks (also commonly termed graphs) [1]. A network consists of a set of nodes (or vertices) and a set of edges [16]. Edges can include varying weights or direction and nodes can have multiple attributes. Different systems can be modeled by different structures of graphs such as directed [17], weighted [18], overlapping and clustered [19], or hierarchical graphs [20].

Unlike random graphs, in which the probability of having an edge between two nodes among all pairs of nodes is equal and the distribution of edges is homogenous [21], real-life networks show a high level of heterogeneity in edge distribution [1]. These heterogeneities are often represented as clusters within which intra-cluster edge density is much higher than inter-cluster edges. These clusters are typically referred to as communities in networks [22] and these two terms are usually used interchangeably. In real-life networks, nodes of communities usually share similar characteristics and/or similar interests (e.g., social networks; [22]) and/or have higher levels of interactions (e.g., transportation networks; [23]). Finding communities in networks can provide new insights into the structure of networks and enable us to better understand the underlying complex system.

Different algorithms have been developed to detect communities from networks. Graph partitioning methods divide the graph into a specific number of communities with a specified number of nodes to minimize the number of inter-community edges [24, 25]. Hierarchical algorithms comprise two types [26]: bottom-up (agglomerative) in which all similar communities are merged until reaching a specified threshold and top-down (divisive) which adhere to the opposite direction–dividing dissimilar communities until reaching a specified threshold. A very well-known divisive community detection method was proposed by Girvan and Newman [22, 27]. In Girvan and Newman’s method, edges are iteratively removed based on their value of betweenness which expresses the frequency of shortest paths between all pairs of nodes that pass along the edge. An alternative set of methods are modularity-based algorithms [28, 29]. Modularity, as a quality measurement of community detection, is computed based on the comparison between the existing structure of edges in the subgraph and the density of edges in the subgraph if the nodes were attached irrespective of the community structure [27, 30]. Spectral algorithms [31], methods based on statistical inferences [32], and algorithms that use deep learning methods [33] are the other alternatives. For a more comprehensive review of clustering and community detection algorithms, refer to [1, 34].

Methods for measuring the similarity (or differences) between two sets of communities have been employed in methodological work to test the performance of a community detection method on a benchmark [35], or to cross-check the results of community detection using different methods [13]. In applied settings, network community similarity is used across various domains such as studying similarity of sets of communities over time [36], and comparing different types of networks [37]. While there is extensive literature on community detection methods, there is a lack of attention to similarity measurements for network communities [1].

There are four primary types of measures for assessing network community similarity: pair counting, community matching, information theory, and distance. Pair counting methods identify corresponding communities in two different sets. If a node is identified in the same community (two communities with the highest overlapping) in two different sets, it is considered correctly detected. The Rand index [4] and its adjusted version [2], the Jaccard index and its adjusted version [2], and the Mirkin metric [3] are all pair counting methods. Community matching measures are based on finding the largest overlap between two sets of communities. However, these methods may only consider the largest similar portion of communities, potentially disregarding certain parts of the community set as a whole. Classification error, defined by Meilă & Heckerman [38], and the normalized Van Dongen metric [5] are examples of community matching measures. A third type of similarity measure is based on information theory [39]. Using information theory, if two sets are alike, there is little information needed to derive one set from the other. Less similarity between the two sets indicates that more information is needed to infer one from the other. A commonly used measure of this type is the normalized mutual information [15]. Lastly, distance-based measures in which the number of movements and divisions are considered to calculate the distance between two sets. Movement is defined as the minimum number of nodes that are needed to be moved from one community to the meet set of two communities to match, and divisions are defined as the number of divisions needed for one community to match with the meet set of two communities [6, 7].

## 3. Network community structure similarity index (NCSSI)

We first define notation that will be used throughout the paper. Let:

*V* be a set of *n* nodes in network *N*

*E* be a set of edges that connect network nodes

*W* be a set of weights associated with each edge

A community set is defined as a set of >1 communities derived from a given network. The goal of NCSSI is to quantify the similarity between two community sets. NCSSI is derived from the edit distance concept in which the distance is computed based on the total cost to transform one community set into another. The critical feature of NCSSI is the cost function which is associated with each of the necessary edits (inserts and removes) to perform this transformation. We should emphasize that in NCSSI, we are not trying to transform one network to another but rather to transform one community set into another. This is an important distinction as transforming the whole structure of the graphs is unnecessary when the principal focus is only on the community structures. Hence, NCSSI focuses on nodes whose labels differ between the two sets. Then, for each of these nodes, we calculate the cost associated with the minimum edits needed to change the community labels. Calculation of NCSSI follows a three-step method outlined below.

### 3.1 Step 1 –Pairing communities

To find the similarity between two community sets, we must find the most similar community pairs in each of these two sets. Since both community sets have different community labels, to find the most similar community pairs, we must calculate the most overlapping communities in each set in terms of both nodes and weighted edges. To calculate this, assume we have two communities *x* and *y*, from the community sets *X* and *Y*, respectively, where the intersection set of their nodes is ∩_*xy*_ and the union set is ∪_*xy*_. We focus on incorporating edge weight information into the measurement of NCSSI. Therefore, we sum all edge weights (*W*) between the nodes in ∩_*xy*_ and ∪_*xy*_ and adjust them by dividing it by the total weights of edges in the network. Since we have two community sets with different configurations regarding their edges and associated weights, we need to calculate the sum of all edge weights for both Networks. Finally, the overlap score (OS) of communities *x* and *y* is defined as the ratio of the sum of all adjusted edge weights in ∩_*xy*_ to ∪_*xy*_:

$$OS_{xy}=\frac{\frac{\sum_{i,j\in \cap_{xy}}W_{ij}^{X}}{\sum_{i,j}W_{ij}^{X}}+\frac{\sum_{i,j\in \cap_{xy}}W_{ij}^{X}}{\sum_{i,j}W_{ij}^{X}}}{\frac{\sum_{i,j\in \cup_{xy}}W_{ij}^{X}}{\sum_{i,j}W_{ij}^{X}}+\frac{\sum_{i,j\in \cup_{xy}}W_{ij}^{Y}}{\sum_{i,j}W_{ij}^{Y}}}$$ (1)

Where *OS*_*xy*_ is the overlap score between communities *x* and *y* from the community sets of *X* and *Y*; ∩_*xy*_ and ∪_*xy*_ are the intersection and union sets of nodes from communities *x* and *y*; and *$W_{ij}^{X}$* and $W_{ij}^{Y}$ are the edge weights between *i* and *j* in the community sets *X* and *Y*, respectively.

To find the paired communities, we need to calculate all the possible OS values between communities of two community sets, resulting in an OS matrix. The unordered bijective pairs are chosen by the maximum OS, meaning each community in a set is paired only with one community from the other set with the highest overlap. It is possible to have different numbers of communities between the two community sets. If, due to the difference in the number of communities in sets, a community (let us assume *x*) is not paired with a community in the other set, the pair will be denoted as (*x*, *ϕ*).

### 3.2 Step 2 –Calculating edit costs of nodes

Based on the definition of communities, the stronger the intra-community links, and the weaker the inter-community links, the stronger the structure of communities. Hence, in calculating NCSSI, we concentrate on the nodes’ labels and the edge weights associated with the nodes.

In this step, we need to calculate the minimum cost associated with the edits needed to transform the node *i*’s membership (Eqs 2 and 3). In Eqs 2 and 3, we assume that we have community set *X*, which contains communities *x*_*1*_ and *x*_*2*_, and *Y*, which contains communities *y*_*1*_ and *y*_*2*_. Additionally, *x*_*1*_ and *y*_*1*_, and, *x*_*2*_ and *y*_*2*_, are paired with each other.


$$EC_{i}^{X}=|\sum_{j\in x_{1}}W_{ij}-\sum_{j\in x_{2}}W_{ij}|$$ (2)

$$EC_{i}^{Y}=|\sum_{j\in y_{1}}W_{ij}-\sum_{j\in y_{2}}W_{ij}|$$ (3)

Where $EC_{i}^{X}$ and $EC_{i}^{Y}$ is the adjusted edit cost of node *i* in the community sets *X* and *Y*, respectively; Within Eq 2, *x*_*1*_ is the community in which *i* is a member; and *x*_*2*_ is the community which is the paired community of the changed community *y*_*2*_; similarly within Eq 3, *y*_*2*_ is the community in which *i* is a member; and *y*_*1*_ is the community which is the paired community of the changed community *x*_*1*_; *j* is a node member of the communities *x*_*1*_, *x*_*2*_, *y*_*1*_, and *y*_*2*_; and *W*_*ij*_ is the weight associated with the edge between nodes *i* and *j*.

These costs reflect the necessary modifications required to transform the membership of a node from one community to another while considering the associated edge weights. However, it is important to note that these costs may not be on the same scale for both community sets *X* and *Y*, as they depend on the specific characteristics of the community sets and the corresponding edge weights. To calculate edit costs on a common scale, it is necessary to adjust them by the sum of intra-community edge weights associated with node *i* and the sum of weights between *i* and paired community nodes. To adjust edit costs, we need to compute the adjustment factors for both community sets *X* and *Y* (Eqs 4 and 5). It should be noted that the adjustment factors include weightings from all nodes irrespective of whether the node’s community membership remains unchanged or changed.


$$AF^{X}=\sum_{i}\left(\sum_{j\in x_{1}}W_{ij}+\sum_{j\in x_{2}}W_{ij}\right)$$ (4)

$$AF^{Y}=\sum_{i}\left(\sum_{j\in y_{1}}W_{ij}+\sum_{j\in y_{2}}W_{ij}\right)$$ (5)

Where *AF*^*X*^ and *AF*^*Y*^ are the adjustment factor associated with community *X* and *Y*, respectively. The notation for *i*, *j*, *x*_*1*_, *x*_*2*_, *y*_*1*_, *y*_*2*_, and *W*_*ij*_ remains the same as previously defined.

By dividing the edit costs by the adjustment factors, we obtain the adjusted edit costs on a common scale for both community sets (Eqs 6 and 7). These adjusted edit costs are bounded between 0 and 1 for each community set, ensuring that both community sets are on the same scale and comparable. It is important to note that the sum of all adjusted edit costs for each community set cannot exceed 1, as they represent the relative transformation costs within the community sets.


$$AEC_{i}^{X}=\frac{EC_{i}^{X}}{AF^{X}}$$ (6)

$$AEC_{i}^{Y}=\frac{EC_{i}^{Y}}{AF^{Y}}$$ (7)

Where $AEC_{i}^{X}$ and $AEC_{i}^{Y}$ are the adjusted edit cost of node *i* in the community sets *X* and *Y*, respectively. The notation for $EC_{i}^{X},EC_{i}^{Y}$, *AF*^*X*^ and *AF*^*Y*^ remains the same as previously defined.

**Fig 1:**
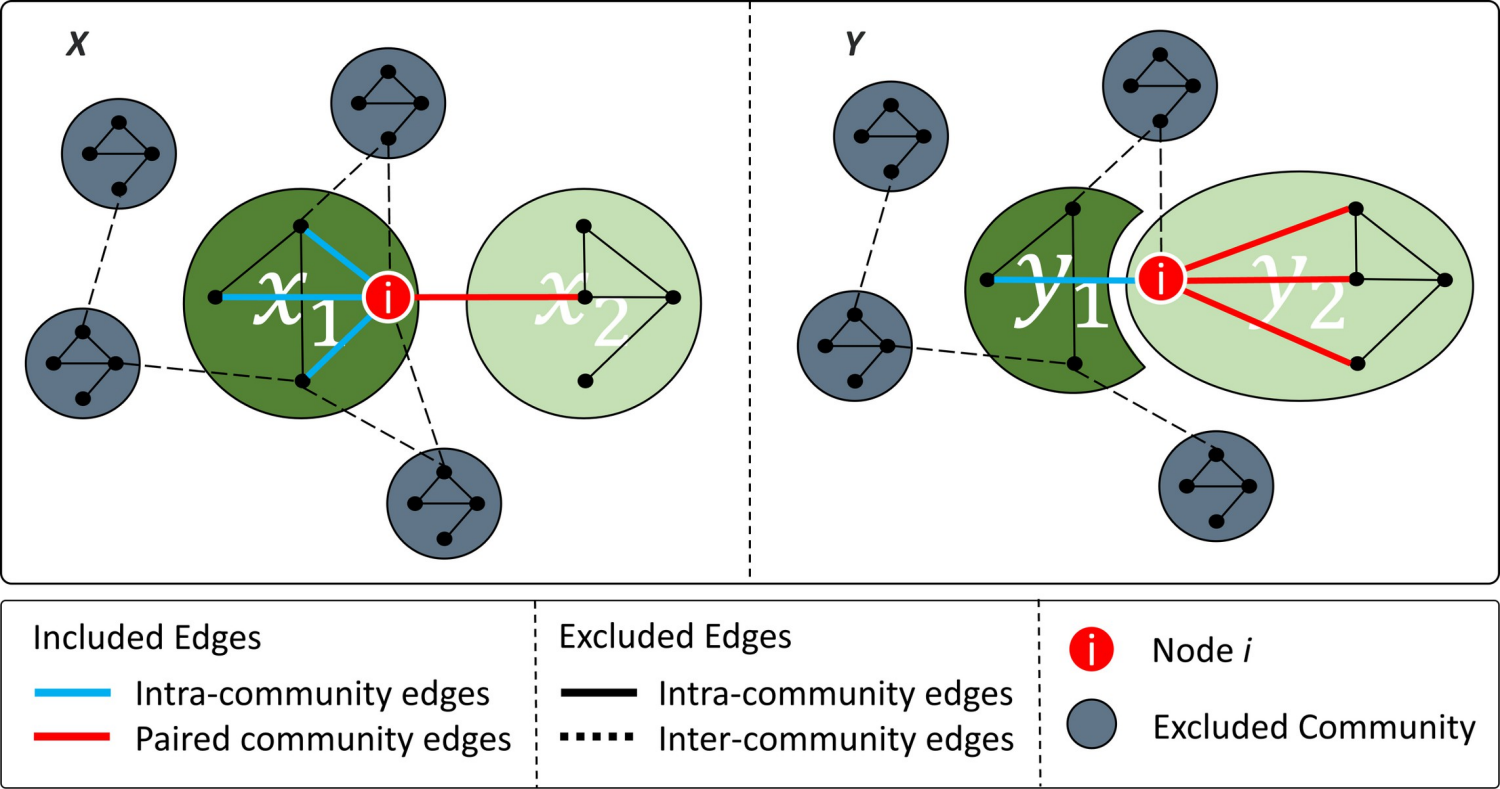
An example of edit cost calculation when node *i* has changed its community membership. Blue lines and red lines represent the edges that are considered in the calculation of the edit cost related to node *i*, as intra-community edges and edges to the paired community. Dashed black lines represent the inter-community edges and black lines represent the intra-community edges that were not considered in the calculation of the edit cost related to node *i*. The paired communities in different community sets are colored the same (dark green and light green). All the other communities that are not considered in the edit cost calculation for node *i*, are colored gray.

As an example, with the same assumption as before, we have community set *X*, which contains communities *x*_*1*_ and *x*_*2*_, and *Y*, which contains communities *y*_*1*_ and *y*_*2*_. Additionally, *x*_*1*_ and *y*_1_, and, *x*_*2*_ and *y*_*2*_, are paired with each other. If node *i* is a member of *x*_*1*_ in community set *X* and a member of *y*_*1*_ in community set *Y*, as the two communities are paired with each other, the node *i* has not changed its community membership—*NEC_i_* is zero. However, if node *i* is a member of *x*_*1*_ in community set *X* and a member of *y*_*2*_ in community set *Y* (Fig 1), as these two communities are not paired with each other, it means that the node has changed its community membership and we need to consider it in our calculation. In this case, in each community set (let us first consider community set *X* and then we must repeat this procedure for community set *Y*) we have two communities that we need to concentrate on; *x*_*1*_ in which *i* is a member, and *x*_*2*_ which is the paired community of the changed community *y*_*2*_ (Fig 1).

### 3.3 Step 3 –NCSSI calculation

NCSSI is the total cost associated with transforming a node’s membership and is equal to the sum of the adjusted edit costs of the node in each of the two sets. Consequently, to get the total cost of transforming one community set into another, we need to sum up all nodes’ *NEC*s from each community set. Then the total cost is obtained by averaging the total adjusted edit costs of both community sets (Eq 8). The selection of the average as the method for combining the adjusted edit costs is justified by several factors. Firstly, taking the average ensures a balanced consideration of both community sets, allowing for equal evaluation of their respective edit costs. Secondly, it preserves the information from both sets by incorporating the collective impact of changes, rather than favoring one set over the other (unlike selecting the minimum or maximum). Thirdly, the average maintains sensitivity to changes in both community sets, capturing the nuanced transformation between the structures. Finally, since the adjustment factors and edit costs are already on the common scale, the average does not introduce bias towards a specific range.


$$NCSSI_{XY}=1-\frac{\sum_{i}AEC_{i}^{X}+\sum_{i}AEC_{i}^{Y}}{2}$$ (8)

**Fig 2:**
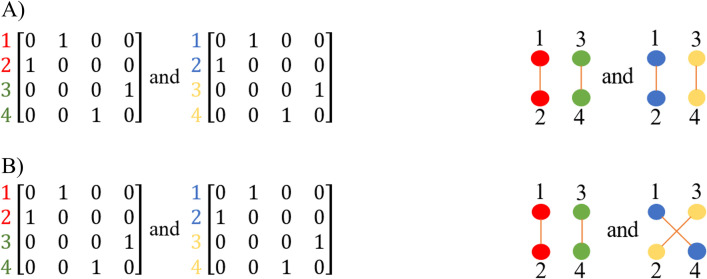
A) An example of identical sets of communities in which community pairs are (red, blue; *OS* = 1) and (green, yellow; *OS* = 1) and *NCSSI* = 1. B). An example of the most dissimilar sets of communities in which community pairs are (red, blue; *OS* = 0) and (green, yellow; *OS* = 0) and *NCSSI* = 0. In these examples, all weights assigned to the edges were 1 for simplicity.

Where *NCSSI*_*XY*_ is the Network Community Structure Similarity Index of community sets *X* and *Y*; *i* is a node from the set of nodes that have changed their community memberships. This results in a value between 0 to 1, which highlights the distance between two community sets. To convert distance to similarity, it is then subtracted from 1. The final value is then defined as the NCSSI of the two sets of communities ranging from 0 to 1 where 0 represents low similarity and 1 identical sets of communities (Fig 2).

## 4. Simulation tests

### 4.1 Benchmark

A benchmark network is a network in which all nodes’ community labels are known. These benchmarks were originally introduced to enable researchers to test the results of community detection methods. Here, we are not interested in testing the clustering and community detection methods, rather, we want to test the community similarity measures using these benchmarks. We use computer-generated benchmarks as they enable us to adjust the network and its attributes with specifically chosen parameters. The most popular class of computer-generated benchmarks works based on *p*_*in*_ as the probability of having intra-community links for nodes and *p*_*out*_ as the probability of having inter-community links while these two probabilities are independent [40]. A specific case of this type was proposed by Girvan and Newman [22] in which a graph with 4 communities and 32 nodes in each community is considered. This benchmark is not an ideal representative of real-life networks as the number of communities and the number of nodes in each community are constant and nodes’ degrees are almost similar in each community.

**Fig 3:**
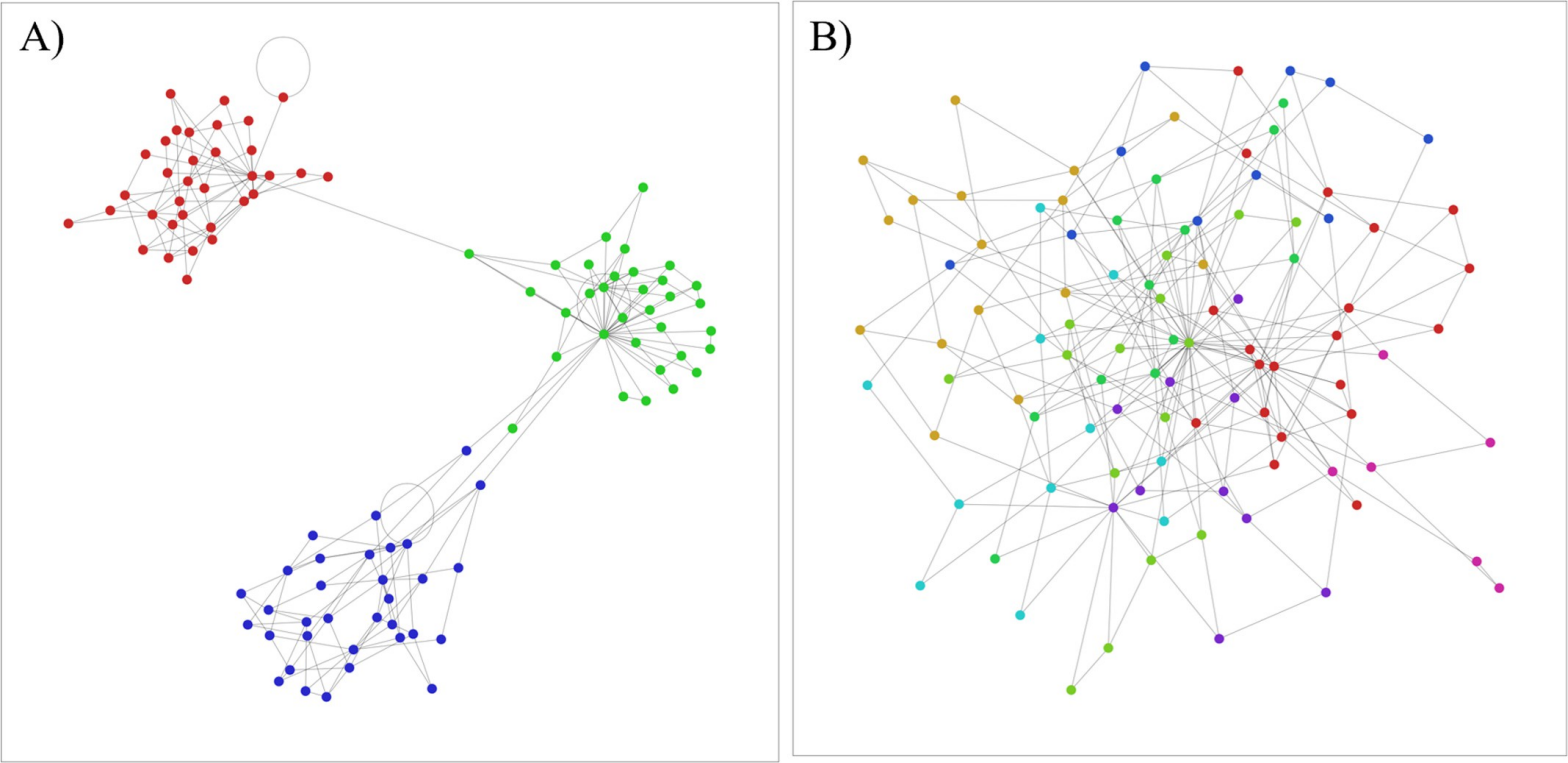
A) LFR benchmark [41] when *μ* equals 0.05, and B) LFR benchmark when *μ* equals 0.95. Each color is representative of the nodes’ communities.

The Lancichinetti-Fortunato-Radicchi (LFR) benchmark introduced by Lancichinetti et al., [41] is a modified version of this model in which the heterogeneity of nodes’ degrees, the community sizes, and the number of communities is taken into account (Fig 3). In the LFR the distribution of nodes’ degrees and the size of communities are determined by power law functions with two parameters of τ_1_ and τ_2_, respectively. The intra-community degrees are determined by the fraction of 1 – *μ* of nodes’ degree. Thus, the inter-community degrees are computed based on the fraction of *μ* of nodes’ degrees. When *μ* is 0 we have highly separated communities but when *μ* is 1 we have highly interconnected communities. Here, we use the LFR benchmark in our experiments.

### 4.2 Different community detection methods

We generated simulated community graphs for comparison using the LFR benchmark. We varied *μ* from 0.1 to 0.95 and for each *μ* we ran the process 100 times as suggested by [41] to avoid random bias. We used *n* = 1000 nodes in the graph and τ_1_ = 1.5 and τ_2_ = 3, as the parameters of the LFR benchmarks. We deployed the Louvain community detection algorithm [42] to derive communities from each benchmark. We computed the similarity index using the Jaccard index, mutual information, and NCSSI by comparing the communities detected by the algorithm to the known benchmark communities. We repeated this process deploying Clauset’s algorithm [28], asynchronous label propagation algorithm [43], and semi-synchronous label propagation algorithm [44] (S1 Appendix).

**Fig 4:**
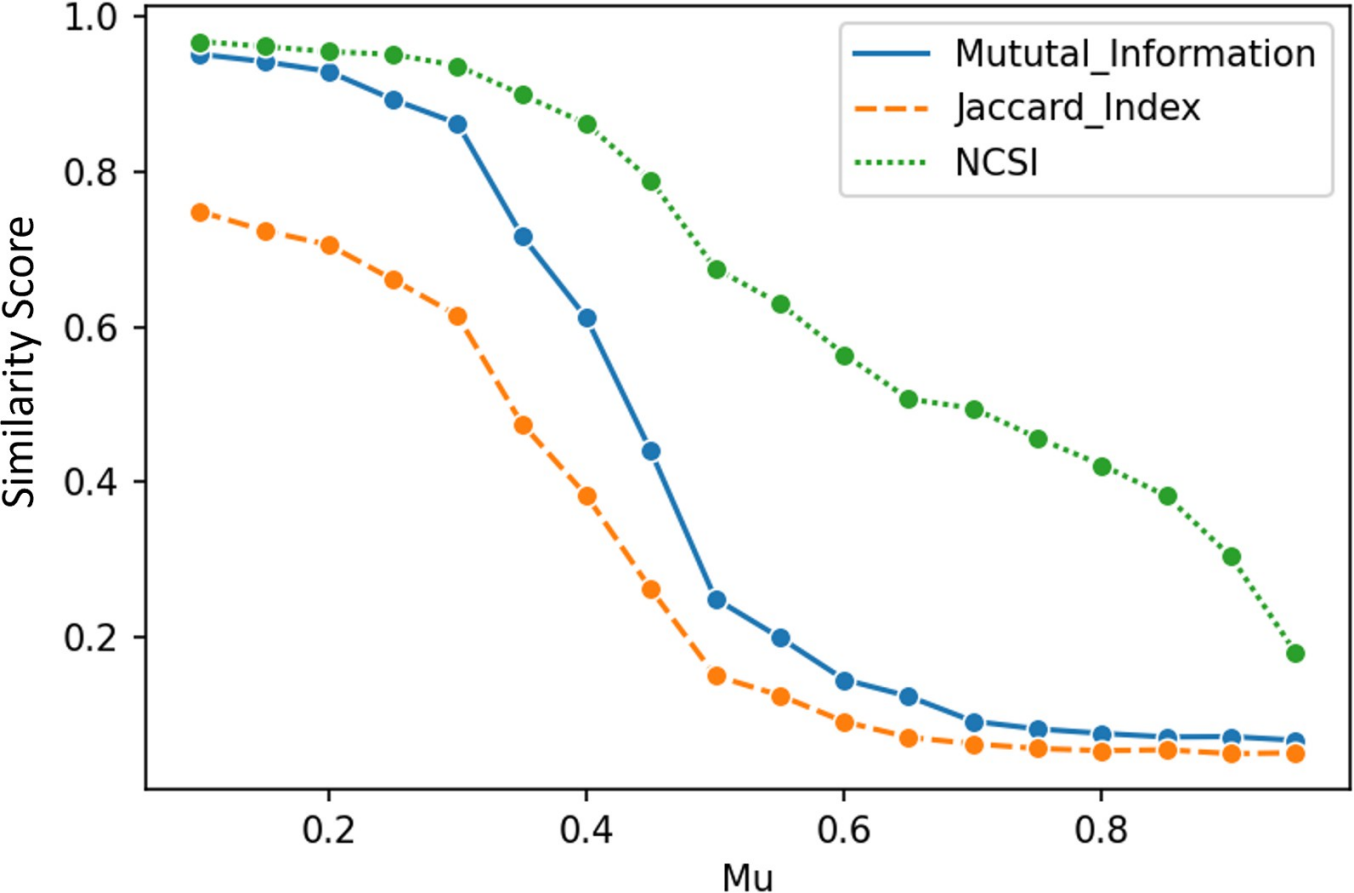
Similarity scores deploying Louvain similarity measure on LFR benchmarks using two existing similarity measurement methods (the Jaccard index and mutual information) and the proposed method NCSSI.

The Jaccard index and mutual information resulted in low similarity between two sets of communities when *μ* > 0.5 (Fig 4). However, considering the network’s structure in the similarity measurement, we observe that increasing *μ* decreases the similarity between known and computed community sets. The relationship between *μ* and NCSSI is more linear and monotonic than with the Jaccard index and mutual information measures. The reason that we still observe a low-level of similarity when *μ* > 0.5, is that even in networks with low community structure, there is an inevitable random degree of similarity across the community sets. Based on LFR benchmark algorithm, when *μ* equals 0.5, the number of inter-links and cumulative intra-links (links with all the other communities) will be equal. Although community structure is commonly perceived to weaken after *μ* = 0.5, it is important to note that in networks with a substantial number of nodes and communities, community structure can still be discernible even when *μ* > 0.5. This is because the presence of a large number of nodes and communities ensures that the intra-links within each community outnumber the inter-links to individual communities. Thus, despite the higher fraction of inter-community edges, the overall network can still exhibit noticeable community structure.

### 4.3 Manually changing nodes’ community memberships and their weights

In this example, we used an LFR benchmark (*n* = 1000, τ_1_ = 1.5, τ_2_ = 3, and μ = 0.2), in which all edges had the same weight of 1. We randomly selected a subset of 10 nodes, which constituted 1% of all nodes. To assess the impact of weights, we increased the weights of all the edges connected to these nodes incrementally from 10 to 100 (with an interval of 10). Then we changed these nodes’ communities as an intervention (intentional misclustering). We aimed to examine how different similarity measures can capture the difference between the communities before and after the intervention. We repeated this process for each weight 100 times and took the mean similarity score of the 100 repetitions.

**Fig 5:**
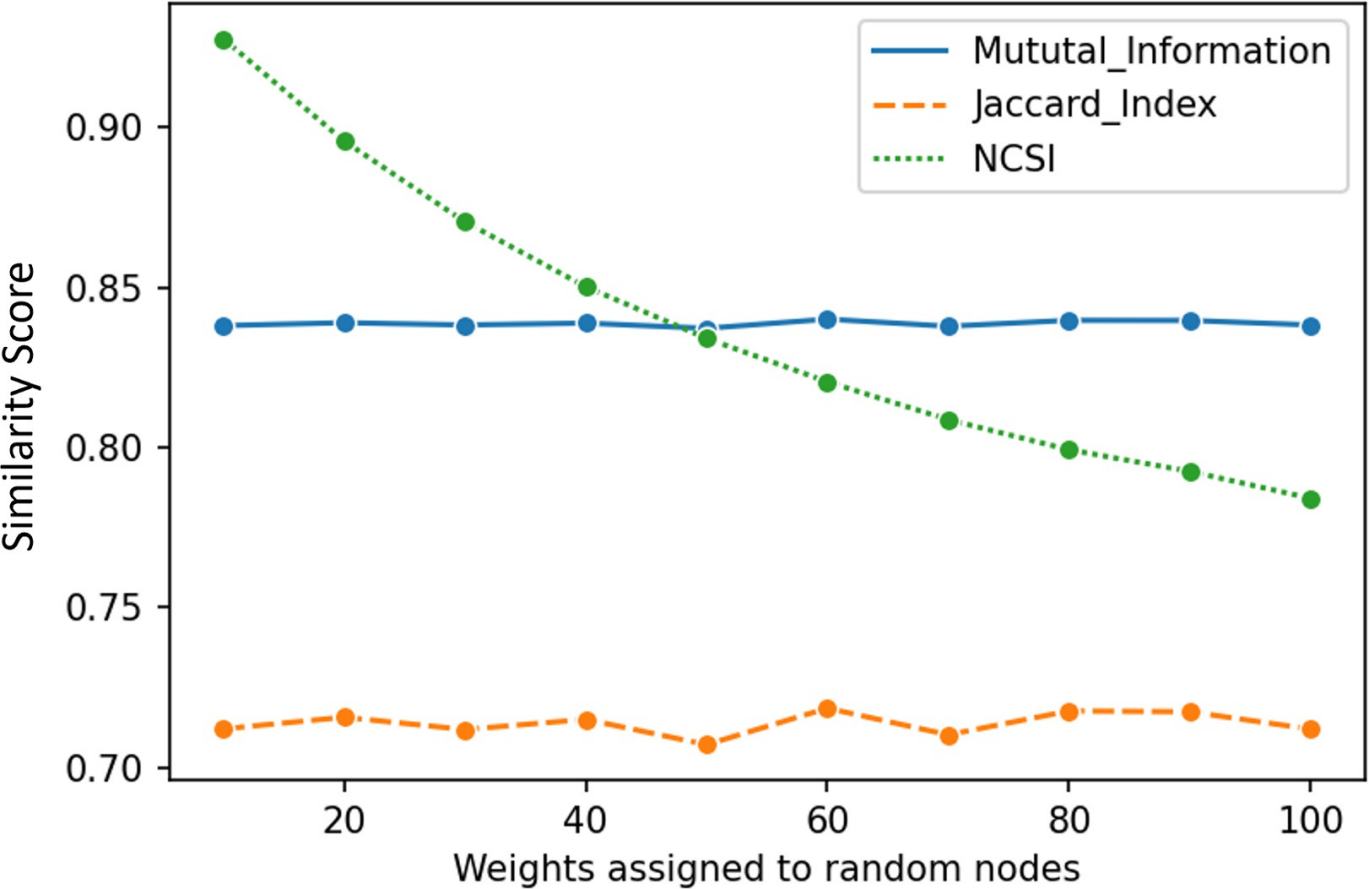
Similarity values for an LFR benchmark while changing the weights associated with 10 randomly chosen nodes using two existing similarity measurement methods (the Jaccard index and mutual information) and the proposed method NCSSI.

The Jaccard index and mutual information measures were not sensitive to the changes in the weights of nodes as increasing the weights associated with these nodes did not affect similarity scores (Fig 5). In contrast, NCSSI captured the effect of increasing weights associated with the misclassified nodes, leading to lower similarity between community sets. These results clearly demonstrate that NCSSI is sensitive to changes in edge weights and the subsequent effect on network community similarity, while Jaccard index and mutual information measures are not.

## 5. Case study: NYC Taxi flow dataset

As a real-life example of how NCSSI can be implemented, we compared how community structures computed based on human mobility flows differ from 2011 to 2021 using Yellow Taxi Pick-up/Drop-off data provided by the New York City Taxi and Limousine Commission, USA [45]. This dataset contains more than 1.3 billion trips for 13,587 taxis in New York City, USA. The locational information in the dataset is aggregated into 263 taxi zones. Each data entry includes a pick-up zone and time, and drop-off zone and time, along with other attributes. We aggregated the data seasonally and created a flow (Origin-Destination) matrix between taxi zones for each season which is a form of a spatial network where zones represent nodes and taxi trip frequencies between zones represent edge weights. We then detected the communities employing Louvain’s algorithm for each season. To avoid seasonal effects, we compared each season’s community structure to the previous year’s. There is no true community structure to compare to, however we can make some hypotheses. First, we do not expect to see community structures change drastically over time, and year to year communities should be relatively stable. However, we do expect to see strong differences associated with the COVID-19 pandemic and associated changes in human mobility patterns in 2019–2020. Further, the advent of ridesharing services (e.g., Uber, Lyft) which originated in 2011 may have an impact over time as they increase in popularity, especially in the less dense areas of NYC. Seven taxi zones appeared to have no trips for at least one season throughout the study period. This causes an inconsistency in the number of nodes in the networks. As mutual information and Jaccard index require the same number of nodes in both networks, this limitation forced us to eliminate inconsistent zones in the networks to compare the results of different methods.

**Fig 6:**
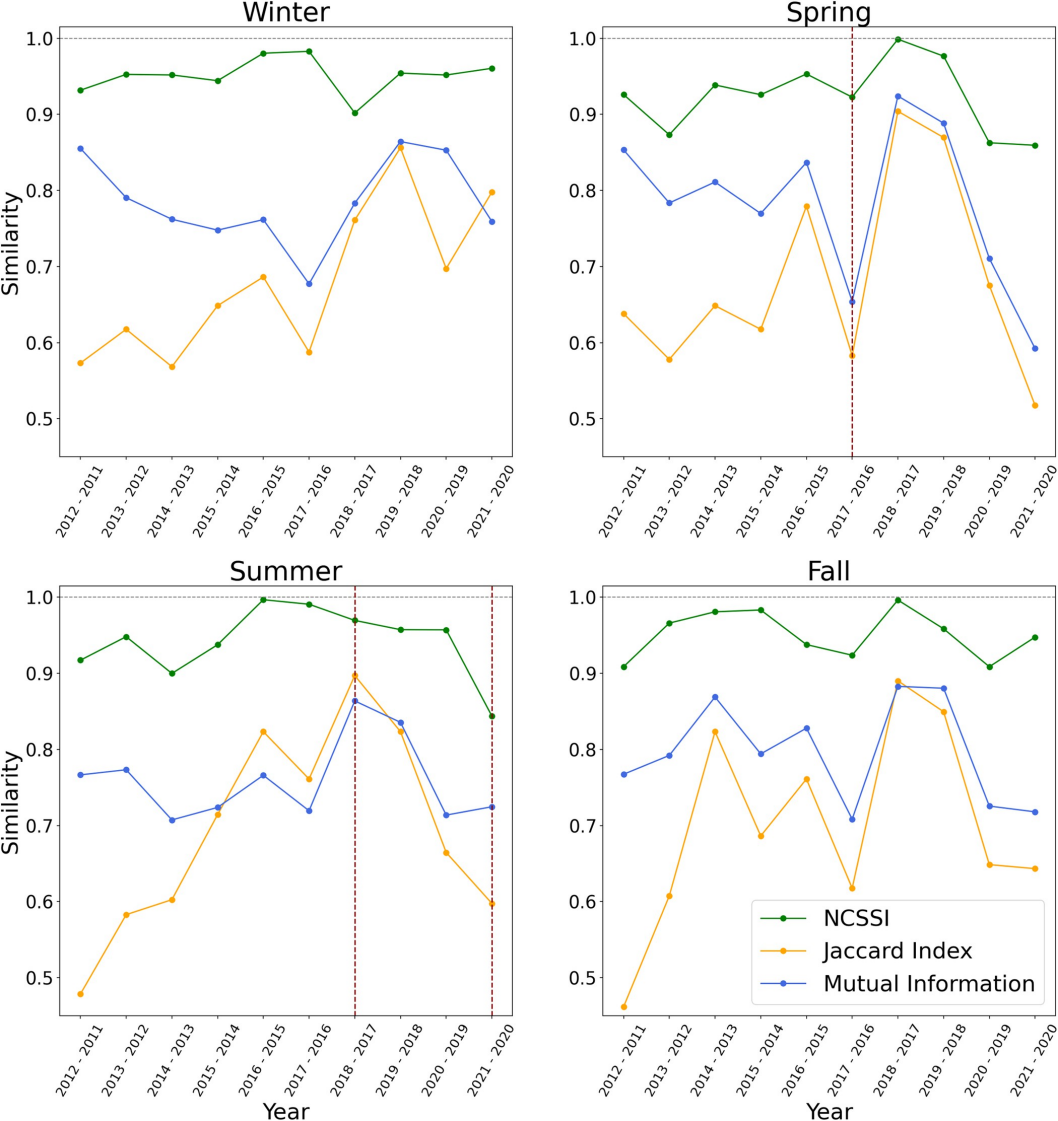
The seasonal level of similarity in community structures for pairs of consecutive years using different indices from 2011 to 2021. The vertical axis demonstrates the similarity score. The dotted red line highlights the three periods of time we discuss in this section (spring 2016–2017; summer 2016–2017; and summer 2020–2021).

We observe a relatively low similarity level between all pairs of consecutive years throughout 2011 to 2021 using the Jaccard index (Fig 6), but this is perhaps not surprising as Jaccard index always presented the lowest similarity between benchmarks. However, the similarity scores of the Jaccard index showed the greatest variation (i.e., lack of consistency in similarity) between consecutive pairs of years (Fig 6). In general, mutual information found higher similarity values relative to Jaccard index (Fig 6), but again showed highly variable results from consecutive pairs of years. NCSSI exhibited the highest similarity values in each pair of years, and more interestingly, NCSSI demonstrated more stable similarity values in all pairs of years leading up to 2019. We see the effect of the COVID-19 pandemic on mobility result in a drop in similarity values in 2019–2020 spring for all measures. In the winter, fall, and spring we see that Jaccard index and mutual information measures show that this similarity continues to be low in the 2020–21 comparisons, but this pattern is not observed using NCSSI.

**Fig 7:**
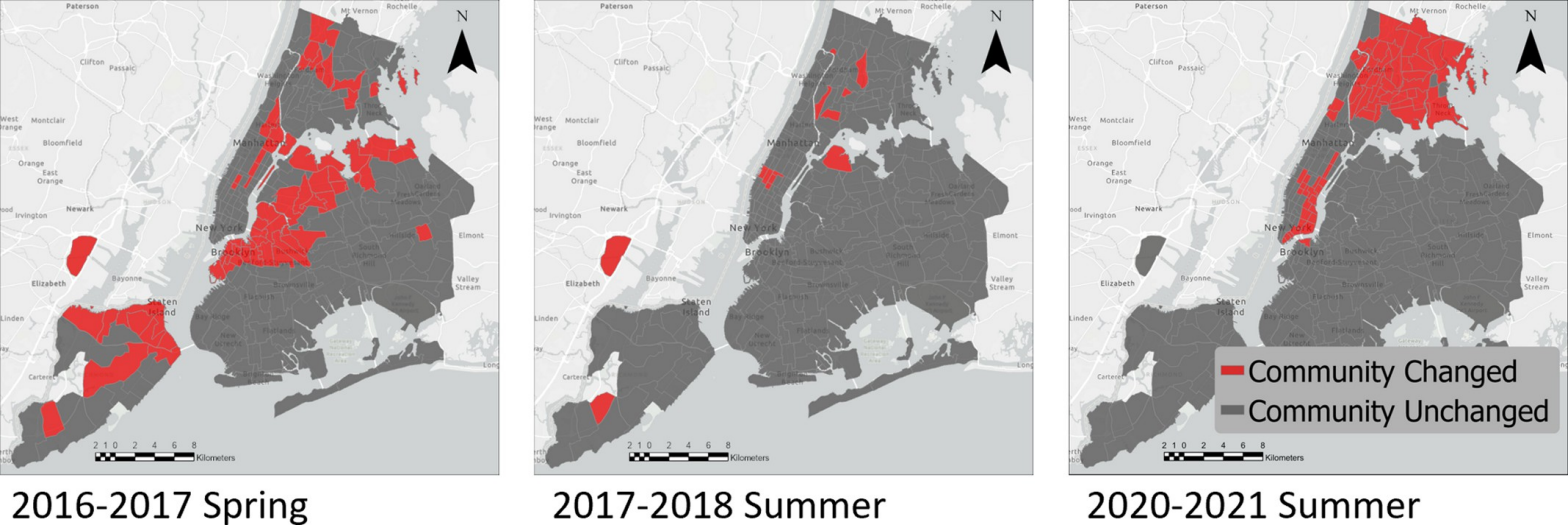
Maps of New York communities computed based on NY Yellow Taxis flow data. Taxi zones colored red represent the areas that changed their communities, and gray zones, represent the areas that did not. Base map and its related data are from OpenStreetMap and OpenStreetMap Foundation [46].

**Fig 8:**
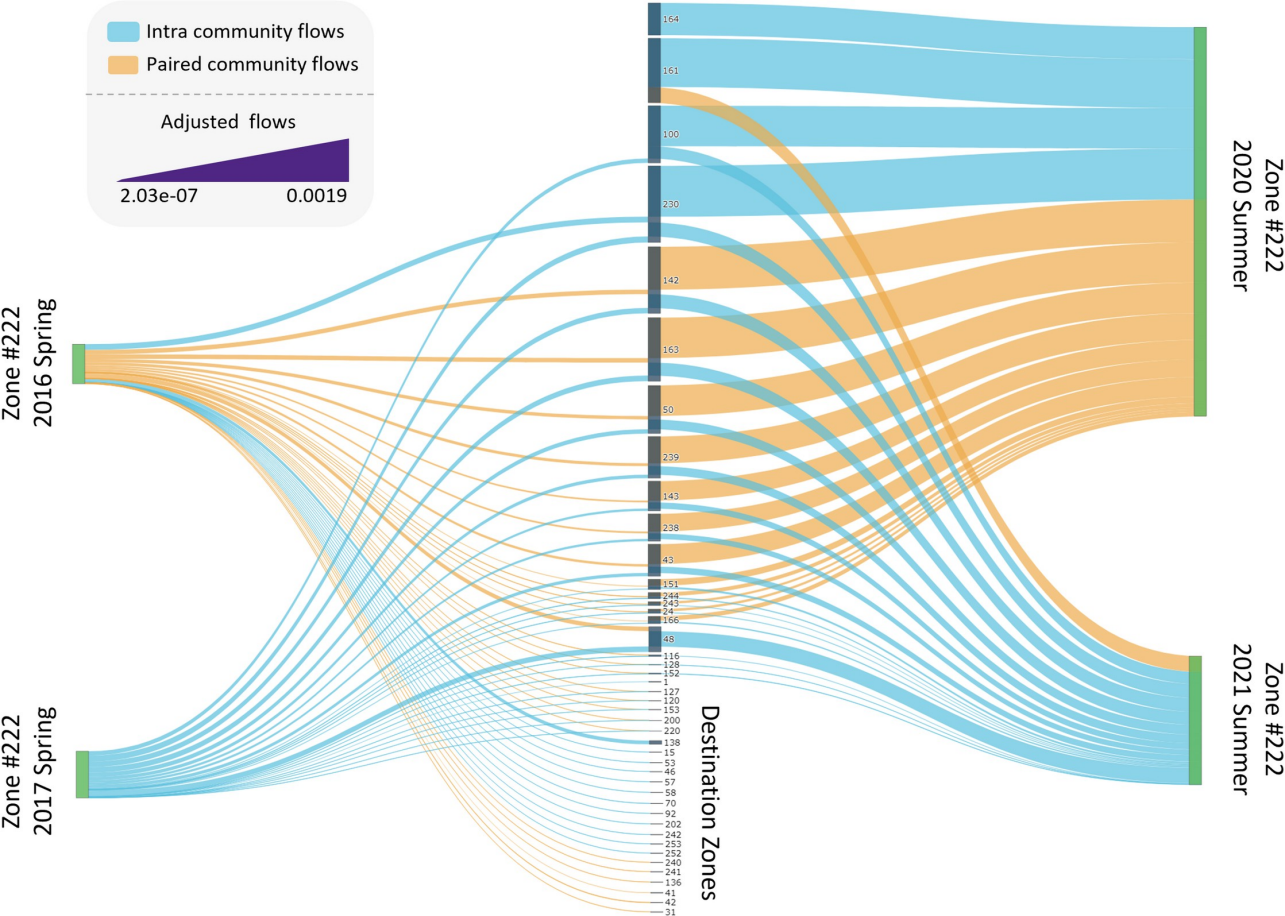
Intra community adjusted flows and adjusted flows to the paired community for taxi zone number 222 for the spring of 2016 and 2017, and the summer of 2020 and 2021. This zone is chosen since it changed its community in all the chosen periods. Intra-community adjusted flows are presented with blue and adjusted flows to the paired community are presented with orange. The width of the flows is based on their intensity; the thicker, the higher rate of adjusted flows.

We chose three periods of time to compare different scenarios and mapped them to visually evaluate the results (Fig 7). The first period is the spring of 2017 compared to the spring of 2018, where the Jaccard index and mutual information resulted in a low level of similarity, but NCSSI showed a high level of similarity. Next is the summer of 2017 compared to the summer of 2018, where all measures demonstrated a high level of similarity. Finally, as an interesting period related to the Covid-19 pandemic, we compared the summer of 2020 to the summer of 2021 where all measures showed a relatively low level of similarity. We observe when the number of zones that changed their communities are higher, as in 2017–2018 Spring and 2020–2021 Summer, the Jaccard index and mutual information resulted in a low level of similarity. However, while the number of communities that changed their community was high, NCSSI did not result in a low level of similarity as the weights of the intra-community flows and flows to the paired community were not substantial in this case, resulting in low edit costs (Fig 8). On the other hand, when the flows substantially changed (as in the summer of 2020–2021), this results in high edit costs, and NCSSI captures a relatively low similarity level (Fig 8).

We plotted intra-community adjusted flows and adjusted flows to paired communities for the spring of 2016 and 2017, and the summer of 2020 and 2021, choosing taxi zone #222, which had a community change in all three periods of interest (Fig 8). It is important to note that the calculated adjusted flows presented in the plots are obtained by dividing the flows by the corresponding adjustment factors associated with each season and year. It is crucial to emphasize that these adjusted flows should be distinguished from actual flows. Despite the fact that the actual flows during the 2020–2021 period experienced a significant decrease, the higher values of adjusted flows compared to the 2016–2017 period is attributed to the flow adjustment process. This process is exclusively employed for the purpose of facilitating visualization, enabling us to visually compare the adjusted flows and providing clarification on how NCSSI incorporates edge weights in its calculations.

We observe that the adjusted flows in the summer of 2020 and 2021 have a higher values relative to the spring of 2016 and 2017. While the number of taxi zones that changed their communities is almost the same in both cases (2016–2017 Spring = 190, and 2020–2021 Summer = 193), the adjusted flow levels differed greatly. For instance, the intra-community and paired community adjusted flows of the zone taxi #222 for the spring of 2016 are 0.003, and 0.141, for the spring of 2017 are 0.017, and 0, for the summer of 2020 are 0.066, and 0.26, and for the summer of 2021 are 0.043, and 0.18 respectively. Since the Jaccard index and mutual information only consider the difference in community memberships and not the flows, they resulted in low values of similarity in both scenarios.

## 6. Discussion and conclusion

In the simulated examples, we changed the fraction of inter-community edges (*μ*) of the benchmarks from 0.1 to 0.95. The Jaccard index and mutual information methods exhibited low similarity after a certain point (*μ* > 0.5) [13], while NCSSI had a more consistent decrease across the range of the fraction of inter-community edges (from 0.1 to 0.95). Therefore, NCSSI seems to be more closely associated with the fraction of inter-community edges than both the Jaccard index and mutual information methods. This observation underscores the intrinsic sensitivity of these methods to changes in network configurations. While the Jaccard index and mutual information methods exhibited diminishing sensitivity when *μ* > 0.5, NCSSI consistently responded across the full *μ* range, capturing variations in edge structures. This behavior highlights NCSSI’s relationship with *μ*, distinguishing it from the Jaccard index and mutual information methods.

In another experiment, we intentionally changed the labels of several nodes and their associated weights to create an intentional difference in the community sets. We observed that Jaccard index and mutual information could capture the differences created by mislabeled nodes, but changing weights did not affect their similarity values as they are primarily sensitive only to changes in the node labels rather than changes in the weights. In contrast, NCSSI demonstrated a distinct advantage in capturing the difference when the weights of intervened nodes change. NCSSI effectively considered both the label and weight information in the communities, enabling it to capture the impact of changes in weights in measuring the similarity between community sets. While there has been considerable focus on integrating weights into community detection methods [42, 47, 48], little attention has been given to incorporating edge weights into similarity measurement methods for community sets. This experiment highlights the distinct advantage of NCSSI over mutual information and the Jaccard index methods, where both label and weight variations are related factors in measuring similarity between community sets.

We employed NCSSI on the New York Yellow Taxi flows dataset as a real-life case study. We computed similarity scores for each season between each pair of consecutive years. Different factors such as technological advancements [49], economic development [50], political and environmental factors [51–53] can influence human communities. The pace of these changes is insufficient [54] to expect major variations in human community structures within two consecutive years. However, we observed a relatively high rate of change in the Jaccard index and mutual information results. On the other hand, NCSSI demonstrated a more stable similarity degree between the communities, except after the outbreak of COVID-19 in early spring 2020.

It is common that entities in complex systems change (established or annihilated) [55]. For example, people might die or be born in social networks, or through policy changes, the number of census tracts might differ over time. While in most cases, community detection is conducted for the same set of nodes [1], there are cases where the number of nodes changes (e.g., social networks, friendship, or professional networks). The NCSSI can handle this without requiring any modifications. To elaborate, when a node is added or removed from one community set to another, the corresponding edge weights connected to that node will also change (added or removed). As NCSSI effectively captures the transformation of community sets by pairing similar communities and calculating the edit costs associated with node membership and their edge weight changes, node additions or removals and consequently edge weights additions and removals will directly impact the NCSSI result. Hence, NCSSI is well-equipped to handle cases where the networks being compared have different numbers of nodes.

In cases when the NCSSI yields high similarity scores for two sets of communities, it is important to recognize that the underlying community set topologies could still differ. It is crucial to emphasize that the NCSSI does not assess the overall similarity of network (graph) structures. Instead, its focus is specifically on quantifying the similarity between the structures of the community sets themselves.

In conclusion, NCSSI offers a new approach to better measure and assess community similarities. By considering both node labels and edge weight variations in communities, NCSSI enhances the accuracy of the similarity measurement between community sets. It also allows measuring similarity between communities with different number of nodes. This capability is particularly valuable where the community sets are of varying sizes and compositions. It is worth noting that NCSSI incorporates the principles of edge importance, weight awareness, and edge submodularity, introduced in [56] primarily for graph similarity measurements, since it effectively captures the impact of edge changes only on the edges associated with nodes that change their communities, acknowledges the significance of edge weights in weighted graphs, and accounts for the varying importance of edge changes in dense and sparse networks.

## Supporting information

S1 Appendix (DOCX)
