# Supplementary material for: A network community structure similarity index for weighted networks
Source: PLoS One. 2023 Nov 29;18(11):e0292018. doi: 10.1371/journal.pone.0292018 (PMC10686481; doi:10.1371/journal.pone.0292018)
Supplement: S1 Appendix — (DOCX) [file pone.0292018.s001.docx]

# S1 Appendix

We computed different similarity score using different similarity methods on LFR benchmarks, as indicated in section 4.2. Here are the results of three different community detection algorithms.


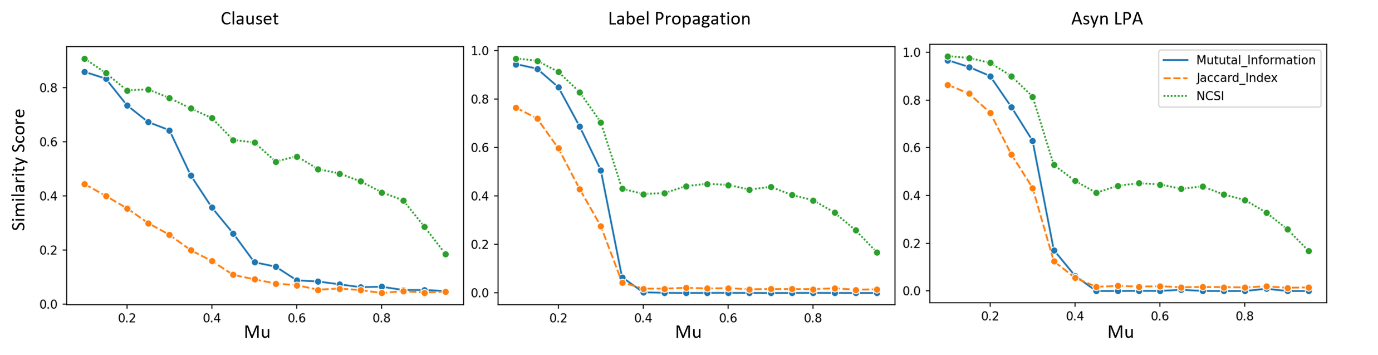


Fig S1-1: Similarity scores of three different community detection algorithms (Clauset's algorithm [28], asynchronous label propagation algorithm [43], and semi-synchronous label propagation algorithm [44]) on LFR benchmarks using three different similarity measurement methods.
